# Supplementary material for: A-MYB and BRDT-dependent RNA Polymerase II pause release orchestrates transcriptional regulation in mammalian meiosis
Source: Nat Commun. 2023 Mar 29;14:1753. doi: 10.1038/s41467-023-37408-w (PMC10060231; doi:10.1038/s41467-023-37408-w)
Supplement: Supplementary file 1 — Supplementary Information [file 41467_2023_37408_MOESM1_ESM.pdf]

Supplementary Information

A-MYB and BRDT-dependent RNA Polymerase II pause release orchestrates transcriptional regulation in mammalian meiosis

Adriana K. Alexander[1,2], Edward J. Rice[1], Jelena Lujic[2], Leah E. Simon[2], Stephanie Tanis[2], Gilad Barshad[1], Lina Zhu[1], Jyoti Lama[2], Paula E Cohen[2,3,*], Charles G. Danko[1,2,3,*]

[1]Baker Institute for Animal Health, College of Veterinary Medicine, Cornell University, Ithaca, NY 14853.
[2]Department of Biomedical Sciences, College of Veterinary Medicine, Cornell University, Ithaca, NY 14853.
[3] Cornell Reproductive Sciences Center (CoRe), Cornell University, Ithaca, NY 14853

Supplementary Figure 1

Supplementary Figure 2

Supplementary Figure 3

Supplementary Figure 4

Supplementary Figure 5

Supplementary Figure 6

Supplementary Figure 7

Supplementary Figure 8

Supplementary Figure 9

Supplementary Figure 1

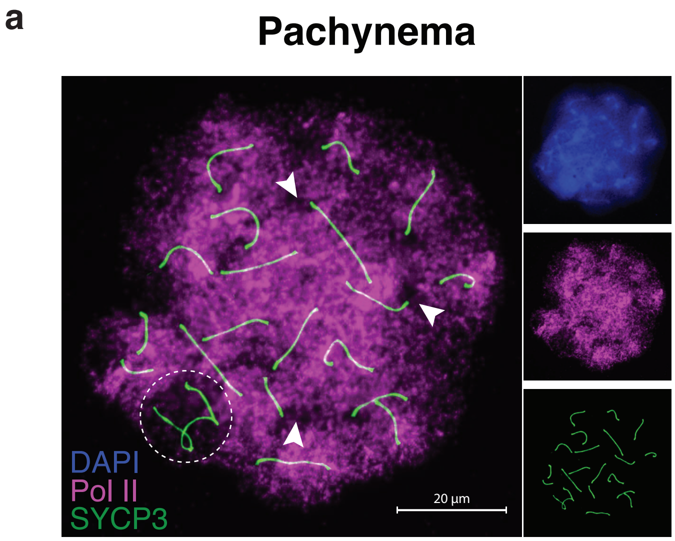

Supplementary Figure 1. **Localization of RNA Pol II on a pachytene spermatocyte chromosome spread. a** Immunolocalization of Pol II (magenta), SYCP3 (green), and DAPI (blue) in WT pachytene spermatocytes. The sex body, housing the X and Y chromosomes, is outlined in white. Arrows indicate absence of Pol II signal near pericentromeric heterochromatin. *n* = 606 prophase I cells.

Supplementary Figure 2

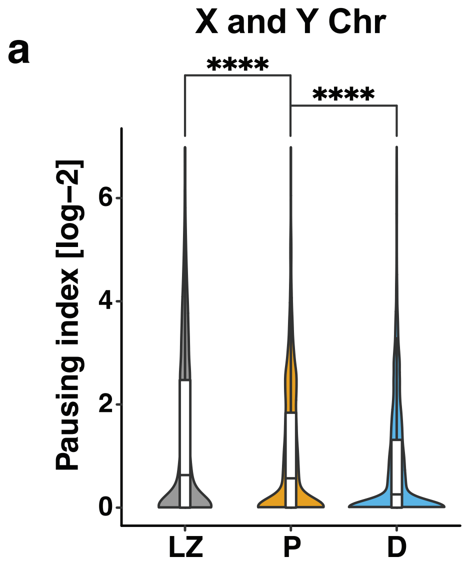

Supplementary Figure 2. **Pausing index on the X and Y chromosomes. a** Violin plot of pause index values calculated from the ratio of pause peak density to gene body density of leChRO-seq reads on the sex chromosomes. ****$p < 0.0001$, two-sided Wilcoxon matched-pairs signed rank test. Median value is indicated by a horizontal black line. The first and third quartile are indicated by the minima and maxima of the boxplot. $n = 4$ biologically independent samples. Source data are provided as a Source Data file.

Supplementary Figure 3

a

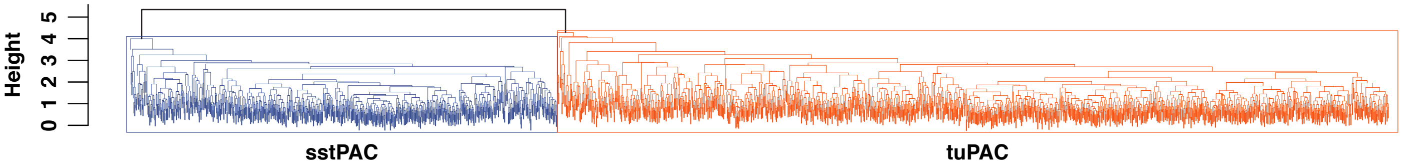

Supplementary Figure 3. **Clustering patterns for sstPAC and tuPAC clusters. a** Dendrogram showing the global hierarchical clustering pattern of genes identified in Figure 3 b and c.

Supplementary Figure 4

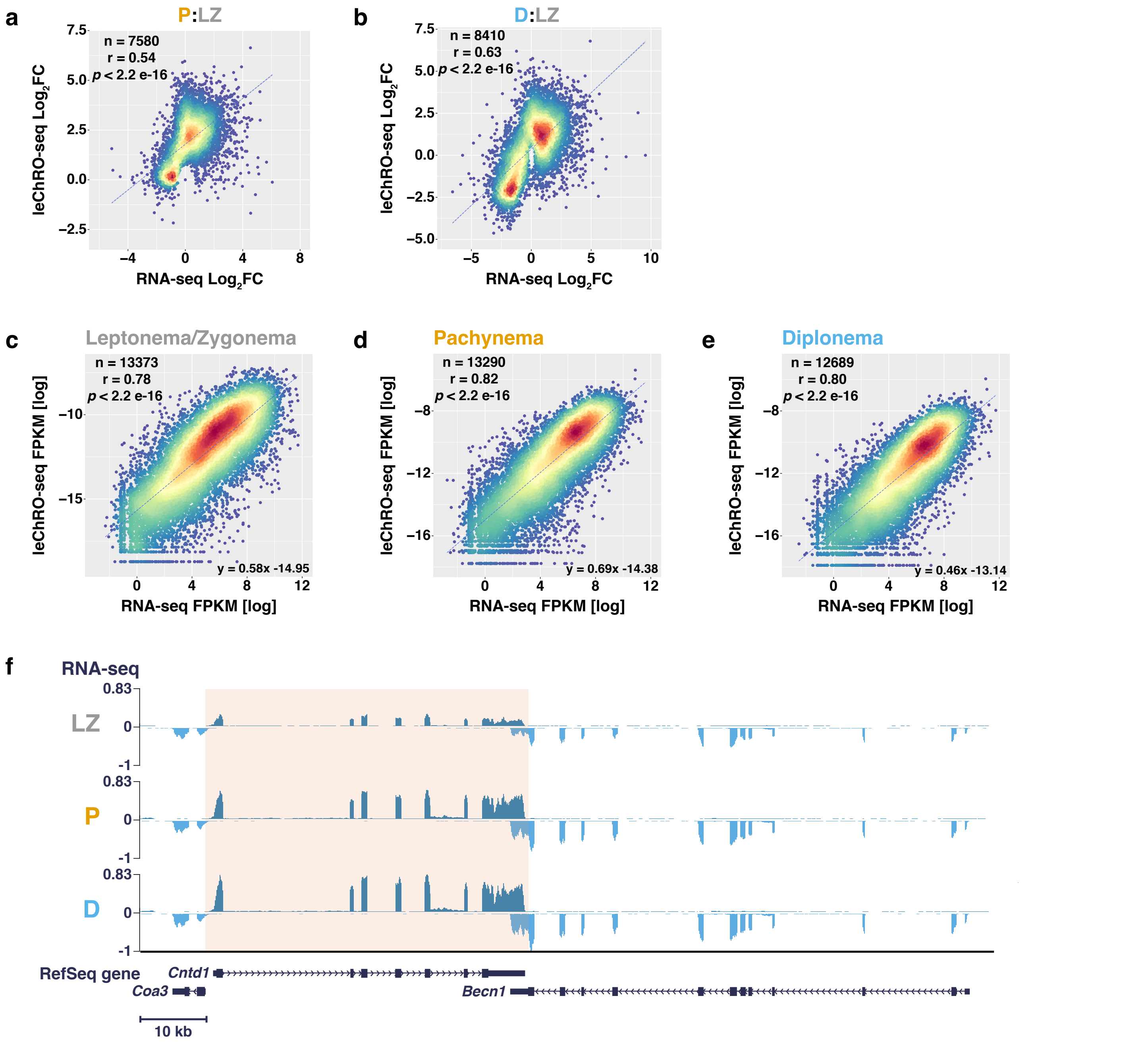

Supplementary Figure 4. **Matched leChRO-seq and RNA-seq libraries are well correlated within individual prophase I substages. a-b** Scatterplots of leChRO-seq vs RNA-seq $\log_2$FC of all differentially expressed genes for pachynema (**a**) and diplonema (**b**) compared to leptonema/zygonema. (See methods section). **c-e** Scatterplots of leChRO-seq vs RNA-seq read counts for differentially expressed annotated genes in prophase I cells shown in units of log fragments per kilobase of transcript per million mapped reads (FPKM). (See Methods section). Panels represent scatterplots of matched leChRO-seq and RNA-seq libraries for leptonema/zygonema (**c**), pachynema (**d**), and diplonema (**e**). For each scatterplot, the Pearson correlation coefficient (*r*), number of differentially expressed genes, and *p*-value are shown. Parallel analyses for each prophase I substage revealed a positive correlation between nascent transcription and mRNA expression, with the highest Pearson correlation coefficient in pachynema. **a-e** Statistical testing was performed with a one-sided t-test. Source data are provided as a Source Data file. **f** Reads per kilobase million (RPKM)-normalized RNA-seq signal at the *Cntd1* locus for leptonema/zygonema (LZ; top), pachynema (P; center), and diplonema (D; bottom). mRNA expression levels are highest in diplonema.

Supplementary Figure 5

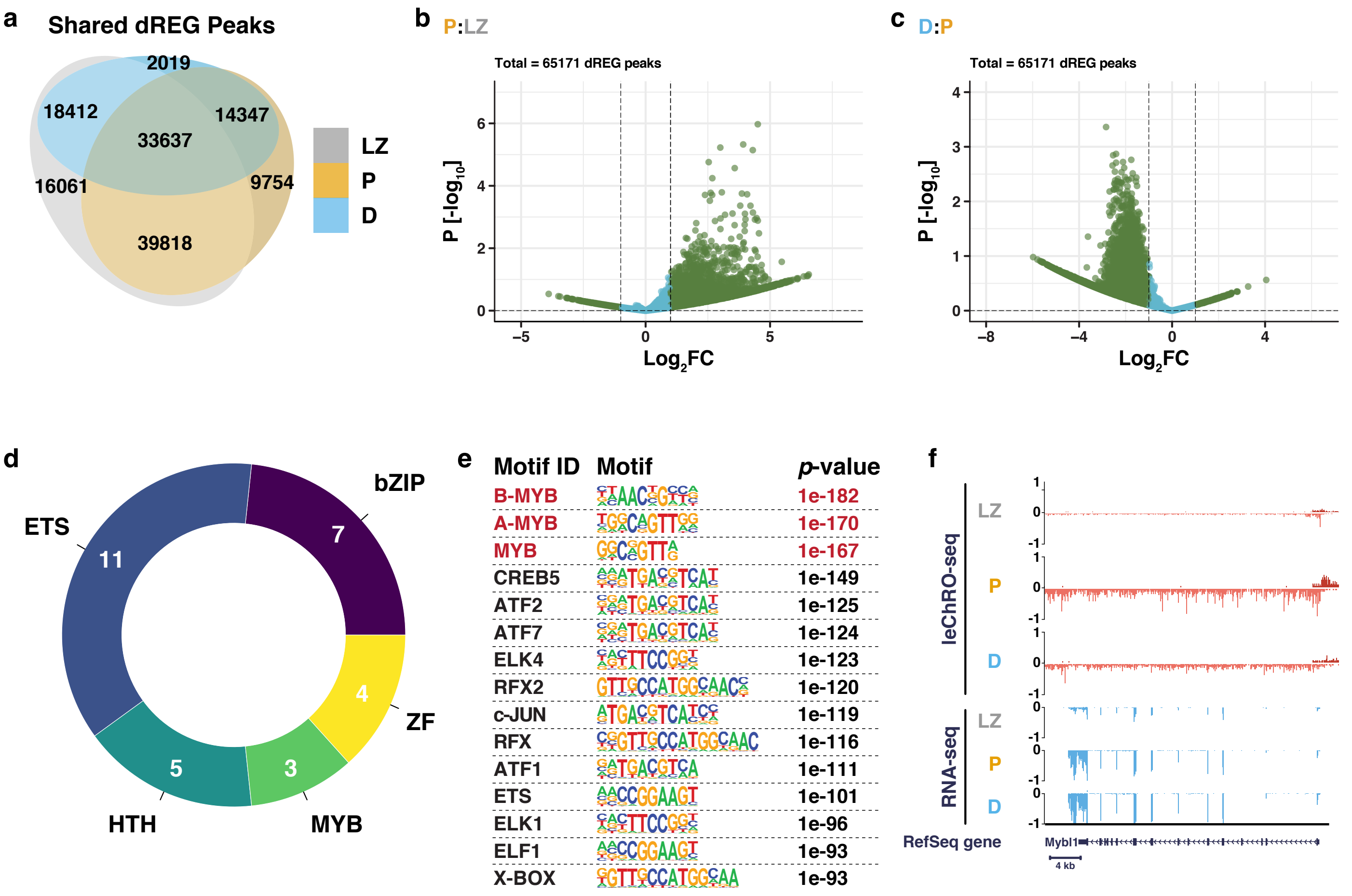

Supplementary Figure 5. **Figure S5. Transcription factors influencing transcriptional activation in pachynema. a** Representative overlap of transcriptional regulatory elements (TREs) identified from dREG by stage. Stage-specific and shared regulatory elements are indicated for each stage of prophase I. Prophase I TREs show considerable overlap between all stages. The number of active TREs decreases throughout prophase I. **b-c** Volcano plots representing DEseq2-based differential expression analysis of dREG-identified TREs comparing leptonema/zygonema and pachynema (**b**) and pachynema and diplonema (**c**). Transcription factor binding motif analysis was performed on the TREs meeting our statistical threshold and are shown in green. Statistical testing was performed with DESeq2, which uses a negative binomial generalized linear model. **d** Donut plot showing the clustering of transcription factor binding motifs based on DNA-binding specificities and transcription factor family. The number of TFs from panel E in each TF family are labeled. **e** Transcription factor binding motifs showing the most significant enrichment in upregulated TREs in pachytene spermatocytes. All motifs shown were identified by HOMER and were significantly enriched with an FDR < 0.01 (one-sided Fisher's Exact Test). **f** leChRO-seq signal and RPKM-normalized RNA-seq signal at the *Mybl1* locus for LZ (top), P (center), and D (bottom). Positive values represent the plus strand and negative values represent the minus strand.

Supplementary Figure 6

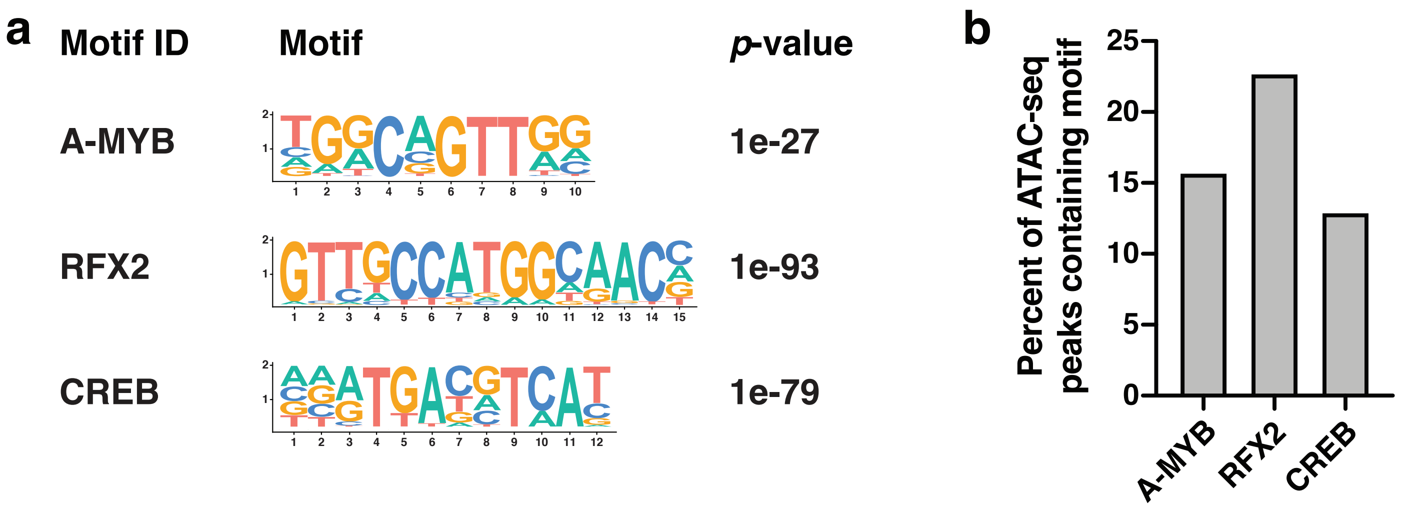

Supplementary Figure 6. **A-MYB, RFX2, and CREB transcription factor binding motif enrichment in ATAC-seq data. a** Transcription factor binding motifs for A-MYB, RFX2, and CREB were significantly enriched in accessible chromatin regions in prophase I spermatocytes. Motifs were identified by HOMER and were significantly enriched with an FDR < 0.01 (one-sided Fisher's Exact Test). **b** Bar graph showing the percent of ATAC-seq peaks containing the motif for A-MYB, RFX2, or CREB.

Supplementary Figure 7

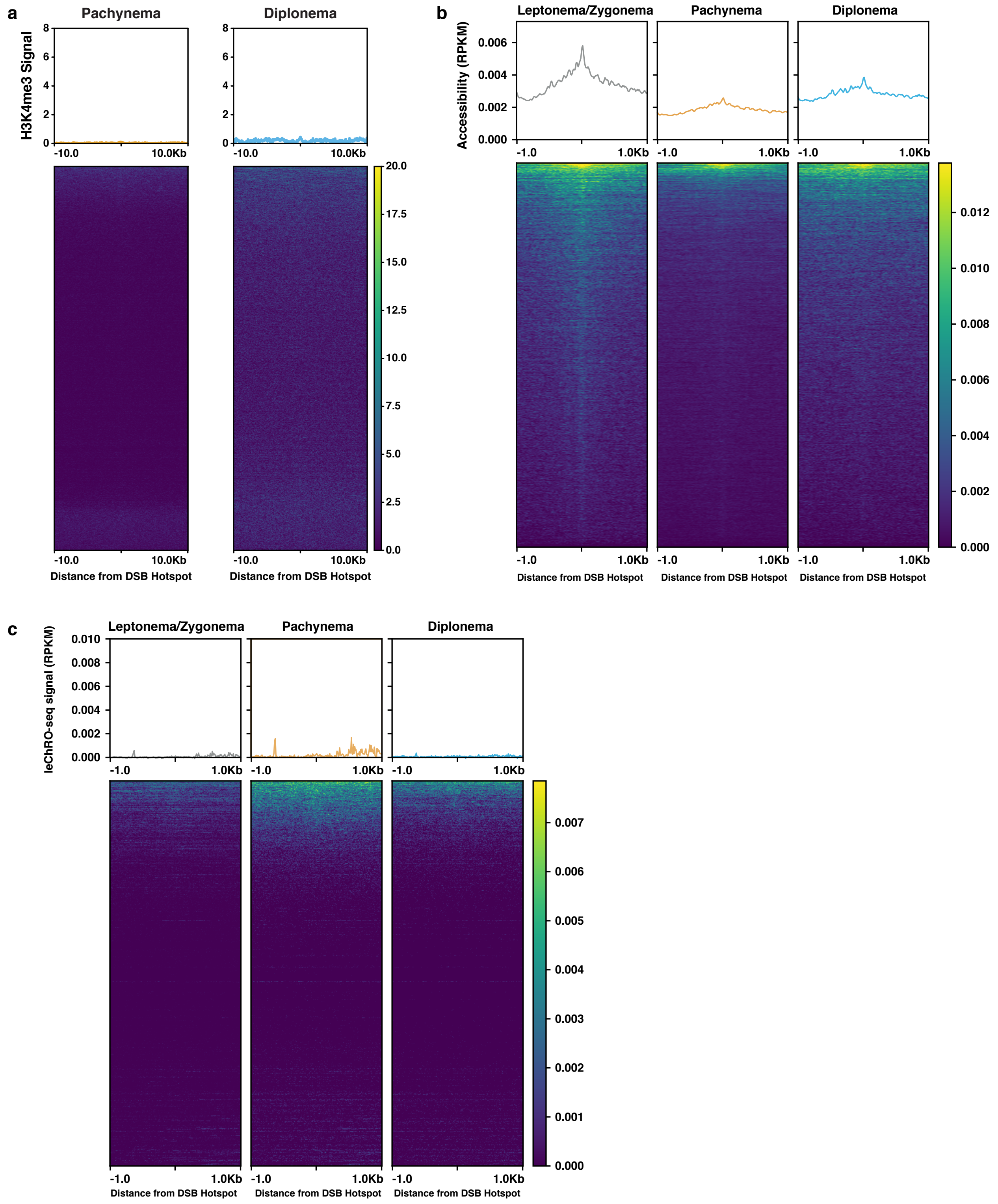

Supplementary Figure 7. **H3K4me3, ATAC-seq, and leChRO-seq signal at DSB hotspots. a** Metaplots (top) and heatmaps (bottom) of H3K4me3 signal centered on DSB hotspots, inferred from SPO11 oligo data, for pachynema and diplonema. **b-c** Metaplots (top) and heatmaps (bottom) of RPKM-normalized ATAC-seq data (**b**) and leChRO-seq data (**c**) centered on PRDM9-independent SPO11 oligo sites for leptonema/zygonema, pachynema, and diplonema.

Supplementary Figure 8

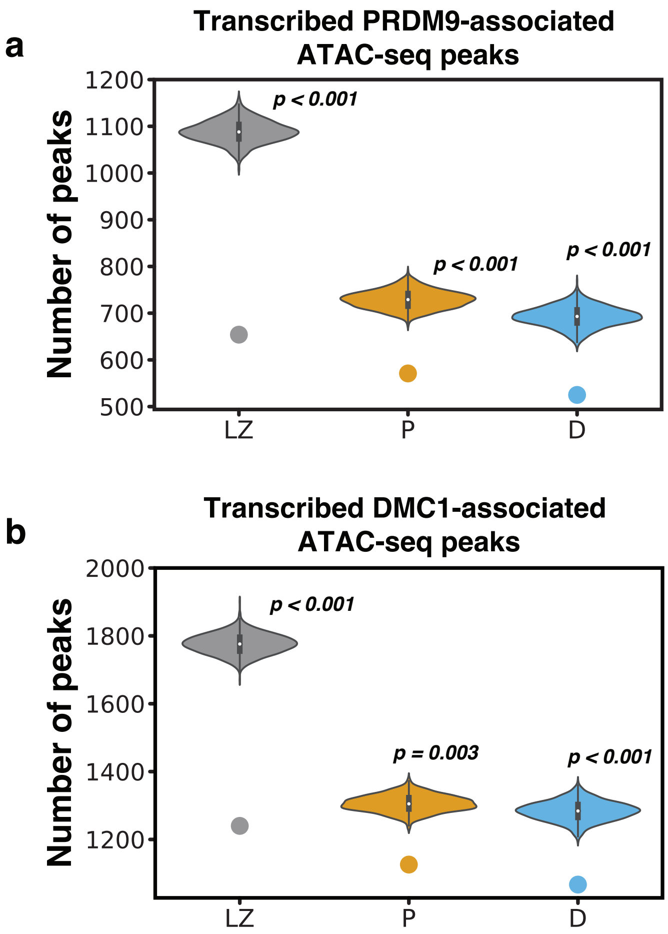

Supplementary Figure 8. **Nascent transcription at PRDM9- and DMC1-associated ATAC-seq peaks. a-b** Observed overlap of PRDM9 motif (**a**) and DMC1 peaks (**b**) with dREG and ATAC-seq peaks (*dot*) and with randomly shuffled dREG and ATAC-seq peaks (*violin plot*). Empirical *p*-value is reported. 15,379 total PRDM9 binding motifs and 30,106 DMC1 ChIP-seq peaks were identified previously(*49*, *53*) and used as input for panels A and B, respectively. Median value is indicated by the white circle in the boxplot. The first and third quartile are indicated by the minima and maxima of the boxplot. Statistical testing was performed with a one-sided Fisher's exact test; $p = 0.000999$, unless otherwise noted. $n = 4$ biologically independent samples. Source data are provided as a Source Data file.

Supplementary Figure 9

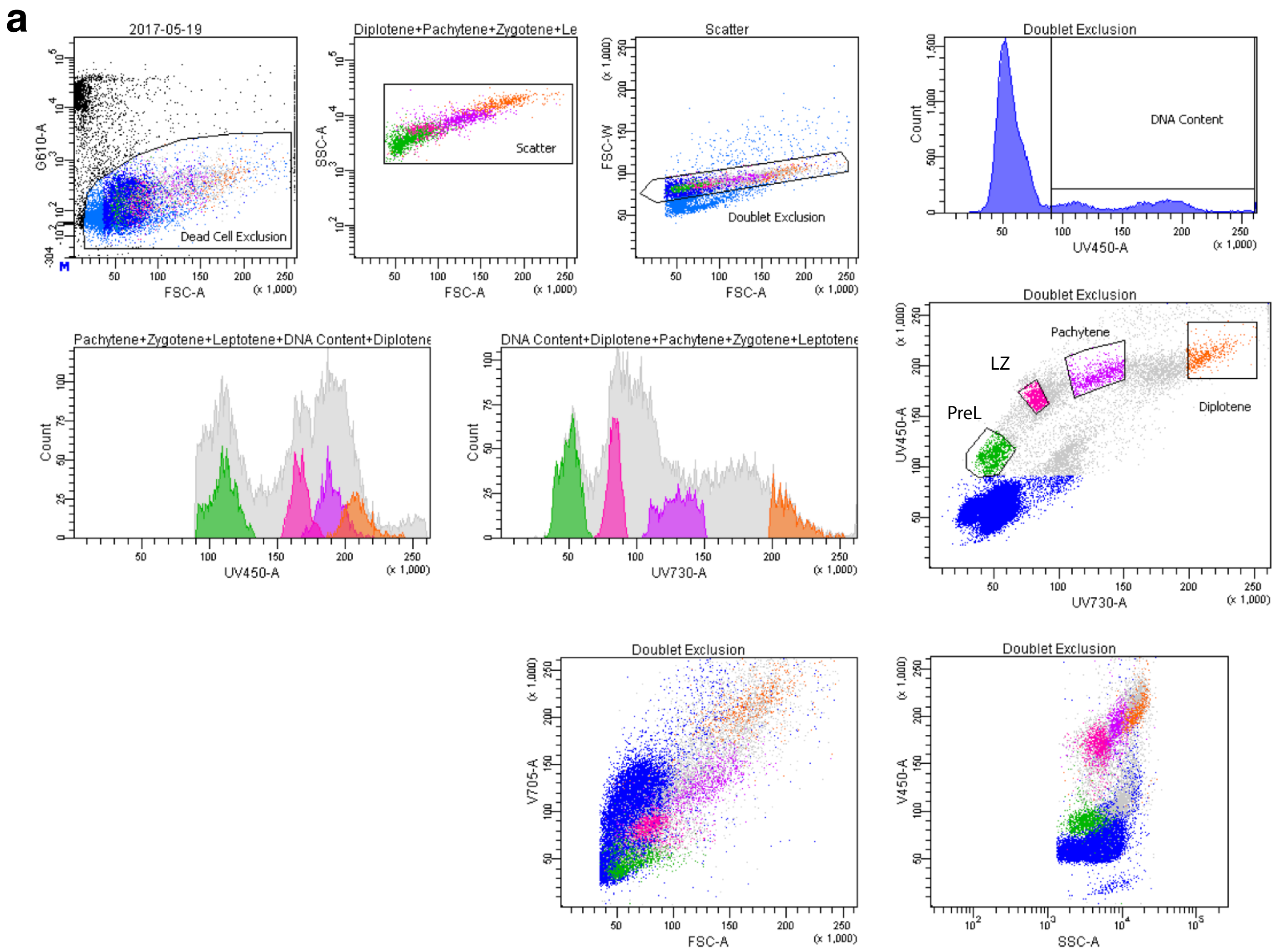

Supplementary Figure 9. **Flow Cytometric Analysis and Fluorescence Activated Cell Sorting of prophase I substages. a** Gating strategy for isolation of prophase I substages by Flow Cytometric Analysis and Fluorescence Activated Cell Sorting (FACS).[69]
