# Supplementary material for: A-MYB and BRDT-dependent RNA Polymerase II pause release orchestrates transcriptional regulation in mammalian meiosis
Source: Nat Commun. 2023 Mar 29;14:1753. doi: 10.1038/s41467-023-37408-w (PMC10060231; doi:10.1038/s41467-023-37408-w)
Supplement: Supplementary file 3 — Reporting Summary [file 41467_2023_37408_MOESM3_ESM.pdf]

nature portfolio

Corresponding author(s): Charles G. Danko

Last updated by author(s): Feb 20, 2023

Reporting Summary

Nature Portfolio wishes to improve the reproducibility of the work that we publish. This form provides structure for consistency and transparency in reporting. For further information on Nature Portfolio policies, see our Editorial Policies and the Editorial Policy Checklist.

Statistics

For all statistical analyses, confirm that the following items are present in the figure legend, table legend, main text, or Methods section.

n/a	Confirmed	
☐	☒	The exact sample size (*n*) for each experimental group/condition, given as a discrete number and unit of measurement
☐	☒	A statement on whether measurements were taken from distinct samples or whether the same sample was measured repeatedly
☐	☒	The statistical test(s) used AND whether they are one- or two-sided<br>*Only common tests should be described solely by name; describe more complex techniques in the Methods section.*
☐	☒	A description of all covariates tested
☐	☒	A description of any assumptions or corrections, such as tests of normality and adjustment for multiple comparisons
☐	☒	A full description of the statistical parameters including central tendency (e.g. means) or other basic estimates (e.g. regression coefficient) AND variation (e.g. standard deviation) or associated estimates of uncertainty (e.g. confidence intervals)
☐	☒	For null hypothesis testing, the test statistic (e.g. *F*, *t*, *r*) with confidence intervals, effect sizes, degrees of freedom and *P* value noted<br>*Give P values as exact values whenever suitable.*
☒	☐	For Bayesian analysis, information on the choice of priors and Markov chain Monte Carlo settings
☐	☒	For hierarchical and complex designs, identification of the appropriate level for tests and full reporting of outcomes
☐	☒	Estimates of effect sizes (e.g. Cohen's *d*, Pearson's *r*), indicating how they were calculated

Our web collection on statistics for biologists contains articles on many of the points above.

Software and code

Policy information about availability of computer code

Data collection	All code used for this manuscript is publicly available at https://github.com/Danko-Lab/Prophase-I-Transcription-Project.
Data analysis	All code used for this manuscript is publicly available at https://github.com/Danko-Lab/Prophase-I-Transcription-Project. Data tools and packages used in the preparation of this manuscript include the following: FIJI v2.9.0, GraphPad Prism v9.0, Zen 2.0 software, FACSAria II, PRINSEQ lite v0.20.4, BWA v0.7.17, mouse reference genome (mm10), BedTools v2.18, Bowtie2 v2.5.1, Genrich v0.6, R v3.4.2, bigWig R package v1.22.0, DEseq2 v1.32.0, R v4.0.5, clust v1.12.0, pheatmap R package in R v4.0.5, dREG v1.0, EnhancedVolcano R package v1.10.0, HOMER v4.11, lessR package v4.0.5, MotifScan v1.3.0, clusterProfiler v4.0.5, Enrichplot v1.12.2, Salmon v1.5.2, tximport v1.20.0, and ggplot2 v3.4.0

For manuscripts utilizing custom algorithms or software that are central to the research but not yet described in published literature, software must be made available to editors and reviewers. We strongly encourage code deposition in a community repository (e.g. GitHub). See the Nature Portfolio guidelines for submitting code & software for further information.

Data

Policy information about availability of data

All manuscripts must include a data availability statement. This statement should provide the following information, where applicable:

- Accession codes, unique identifiers, or web links for publicly available datasets
- A description of any restrictions on data availability
- For clinical datasets or third party data, please ensure that the statement adheres to our policy

Complete leChRO-seq, ATAC-seq, and RNA-seq data generated in this study have been deposited in the NCBI GEO database under accession code GSE212120 [https://www.ncbi.nlm.nih.gov/geo/query/acc.cgi?acc=GSE212120] and are publicly available. PRDM9 motifs and Spo11 oligo data are available at GEO with accession code, GSE84689 [https://www.ncbi.nlm.nih.gov/geo/query/acc.cgi?acc=GSE84689]. DMC1 ChIP-seq peaks are available at GEO with accession code, GSE35498 [https://www.ncbi.nlm.nih.gov/geo/query/acc.cgi?acc=GSE35498]. Reference genome was mm10 (https://www.ncbi.nlm.nih.gov/assembly/GCF_000001635.20/).

Human research participants

Policy information about studies involving human research participants and Sex and Gender in Research.

Reporting on sex and gender	No human research participants were included in our studies.
Population characteristics	NA
Recruitment	NA
Ethics oversight	NA

Note that full information on the approval of the study protocol must also be provided in the manuscript.

Field-specific reporting

Please select the one below that is the best fit for your research. If you are not sure, read the appropriate sections before making your selection.

☒ Life sciences ☐ Behavioural & social sciences ☐ Ecological, evolutionary & environmental sciences

For a reference copy of the document with all sections, see nature.com/documents/nr-reporting-summary-flat.pdf

Life sciences study design

All studies must disclose on these points even when the disclosure is negative.

Sample size	No statistical method was used to predetermine sample size. All ATAC-seq, leChRO-seq, and RNA-seq libraries had 2-4 replicates of 10 mice each. Results were consistent across replicates.
Data exclusions	No data were excluded from analyses.
Replication	All attempts at replication were successful. All genomic assays had 2-4 replicates. All antibody staining measurements used cells from at least 3 mice for analyses.
Randomization	The experiments were not randomized.
Blinding	The experiments were not blinded since no humans were used in this study.

Reporting for specific materials, systems and methods

We require information from authors about some types of materials, experimental systems and methods used in many studies. Here, indicate whether each material, system or method listed is relevant to your study. If you are not sure if a list item applies to your research, read the appropriate section before selecting a response.

Materials & experimental systems

n/a	Involved in the study
☐	☒ Antibodies
☐	☒ Eukaryotic cell lines
☒	☐ Palaeontology and archaeology
☐	☒ Animals and other organisms
☒	☐ Clinical data
☒	☐ Dual use research of concern

Methods

n/a	Involved in the study
☒	☐ ChIP-seq
☒	☐ Flow cytometry
☒	☐ MRI-based neuroimaging

Antibodies

Antibodies used

Antibodies used in this manuscript include those that bind to SYCP3 (custom-made) , RNA Pol II (Millipore Sigma #05-623), RNA Pol II Ser2P (Millipore Sigma # 04-1571), RNA Pol II Ser5P (Millipore Sigma #04-1572), and A-MYB (Sigma Prestige Antibodies #HPA-008).

Validation

All primary antibodies were validated in our lab by comparing staining with and without primary antibody and with secondary antibody in both instances. All commercial antibodies were also validated by commercial providers.

RNA Pol II (Millipore Sigma #05-623): "Anti-RNA polymerase II Antibody, clone CTD4H8 is a high quality Mouse Monoclonal Antibody for the detection of RNA polymerase II and has been published in more than 40 citations and validated for use in ChIP & WB." Source: https://www.emdmillipore.com/US/en/product/Anti-RNA-polymerase-II-Antibody-clone-CTD4H8,MM_NF-05-623

RNA Pol II Ser2P (Millipore Sigma # 04-1571): "Anti-RNA polymerase II subunit B1 (phospho CTD Ser-2) Antibody, clone 3E10 is a Rat monoclonal antibody for detection of RNA polymerase II subunit B1 (phospho CTD Ser-2) has been validated in WB, ELISA." Source: https://www.emdmillipore.com/US/en/product/Anti-RNA-polymerase-II-subunit-B1-phospho-CTD-Ser-2-Antibody-clone-3E10,MM_NF-04-1571

RNA Pol II Ser5P (Millipore Sigma #04-1572): "Anti-RNA polymerase II subunit B1 (phospho-CTD Ser-5) Antibody, clone 3E8 is a highly specific rat monoclonal antibody, that targets RNA Polymerase & has been tested in western blotting & ELISA." Source: https://www.emdmillipore.com/US/en/product/Anti-RNA-polymerase-II-subunit-B1-phospho-CTD-Ser-5-Antibody-clone-3E8,MM_NF-04-1572-I

A-MYB (Sigma Prestige Antibodies #HPA-008): "Anti-MYBL1 antibody produced in rabbit, a Prestige Antibody, is developed and validated by the Human Protein Atlas (HPA) project . Each antibody is tested by immunohistochemistry[3][4] against hundreds of normal and disease tissues. These images can be viewed on the Human Protein Atlas (HPA) site by clicking on the Image Gallery link. The antibodies are also tested using immunofluorescence and western blotting." Source: https://www.sigmaaldrich.com/US/en/product/sigma/hpa008791

Eukaryotic cell lines

Policy information about cell lines and Sex and Gender in Research

Cell line source(s)

Jurkat, Clone E6-1 ATCC Catalog #TIB-152, human cells, male

Authentication

None of the cell lines were authenticated.

Mycoplasma contamination

None of the cell lines were tested for mycoplasma contamination.

Commonly misidentified lines (See ICLAC register)

None of the cell lines were commonly misidentified lines.

Animals and other research organisms

Policy information about studies involving animals; ARRIVE guidelines recommended for reporting animal research, and Sex and Gender in Research

Laboratory animals

The experiments described herein used 10-week-old mice on the C57Bl/6J background and 7-week-old mice on the DBA/2J background, obtained from Jackson Laboratories.

Wild animals

The study did not use any wild animals.

Reporting on sex

Only male animals were included in this study because the subject area was male prophase I of spermatogenesis.

Field-collected samples

The study did not use field-collected samples.

Ethics oversight

All mouse studies were conducted with prior approval by the Cornell Institutional Animal Care and Use Committee, under protocol 2004-0063.

Note that full information on the approval of the study protocol must also be provided in the manuscript.
